# The lipoprotein lipase gene in combined hyperlipidemia: evidence of a protective allele depletion

**DOI:** 10.1186/1476-511X-5-19

**Published:** 2006-07-05

**Authors:** Shu-Fen Wung, Medha V Kulkarni, Clive R Pullinger, Mary J Malloy, John P Kane, Bradley E Aouizerat

**Affiliations:** 1 College of Nursing, University of Arizona, Tucson, AZ, USA; 2 Department of Physiological Nursing, School of Nursing, University of California San Francisco, San Francisco, CA, USA; 3 Cardiovascular Research Institute, University of California San Francisco, San Francisco, CA, USA; 4 Center for Human Genetics, University of California San Francisco, San Francisco, CA, USA

## Abstract

**Background:**

Lipoprotein Lipase (LPL), a key enzyme in lipid metabolism, catalyzes the hydrolysis of triglycerides (TG) from TG-rich lipoproteins, and serves a bridging function that enhances the cellular uptake of lipoproteins. Abnormalities in LPL function are associated with pathophysiological conditions, including familial combined hyperlipidemia (FCH). Whereas two LPL susceptibility alleles were found to co-segregate in a few FCH kindred, a role for common, *protective *alleles remains unexplored. The LPL Ser447Stop (S447X) allele is associated with anti-atherogenic lipid profiles and a modest reduction in risk for coronary disease. We hypothesize that significant depletion of the 447X allele exists in combined hyperlipidemia cases versus controls. A case-control design was employed. The polymorphism was assessed by restriction assay in 212 cases and 161 controls. Genotypic, allelic, and phenotypic associations were examined.

**Results:**

We found evidence of significant allelic (447X_control_: 0.130 vs. 447X_case_: 0.031, χ^2 ^= 29.085; 1df; p < 0.001) and genotypic association (SS: 0.745 vs. 0.939, and SX+XX: 0.255 vs. 0.061) in controls and cases, respectively (χ^2 ^= 26.09; 1df; p < 0.001). In cases, depletion of the 447X allele is associated with a significant elevation in very-low-density lipoprotein cholesterol (VLDL-C, p = 0.045). Consonant with previous studies of this polymorphism, regression models predict that carriers of the 447X allele displayed significantly lower TG, low-density lipoprotein cholesterol (LDL-C) and TG/high-density lipoprotein cholesterol (HDL-C) ratio.

**Conclusion:**

These findings suggest a role for the S447X polymorphism in combined hyperlipidemia and demonstrate the importance of evaluating both susceptibility and protective genetic risk factors.

## Background

Abnormalities of lipoprotein metabolism play an important role in the development of atherosclerosis [1,2], and was one of the earliest established biochemical links to cardiovascular disease [3]. It is well established that elevated plasma triglycerides (TG) are an independent risk factor for coronary heart disease (CHD), and not simply a marker of an inverse relationship with high density lipoprotein-cholesterol (HDL-C), also an independent risk factor for disease [4]. The relative risk of CHD associated with a 1-mmol/L increase in TG was 1.32 (95% CI: 1.26–1.39) in men and 1.76 (95% CI: 1.5–2.07) in women [4]. Thus, identifying factors that determine plasma TG is desirable.

The contribution of lipoprotein lipase (LPL) in the development of dyslipidemia and atherosclerosis is increasingly recognized [5-7]. Numerous studies have shown that LPL is a key enzyme that plays a central role in lipoprotein metabolism and transport [8] and substantial evidence suggests that LPL has an important influence on TG levels [9]. In addition, LPL possesses a non-enzymatic bridging function, assisting in cellular lipoprotein uptake [10]. The finding of triglyceride-rich lipoproteins (TRL) in human atheromata [11,12] has provided substantial pathophysiologic evidence for a direct role of LPL in atherogenesis. Importantly, hereditary forms of dyslipidemia have been in part attributed to ablative mutations in LPL [13].

Though over 200 coding mutations within the LPL gene (MIM 609708) have been described, the role for common functional polymorphism of LPL, particularly in familial combined hyperlipidemia (FCH [MIM 144250]), remains poorly explored [14,15]. The most common amino acid change in LPL is Ser447X (rs328; formally p.Ser474X, c.1421C>G; but throughout the present report we have kept to the traditional nomenclature for this polymorphism), resulting in an LPL protein truncated by two amino acids [16]. It appears likely that the functional properties of the 447X truncated LPL protein is an enhanced bridging function, leading to increased clearance of TRL from the circulation [5]. Interestingly, recent cohort studies suggest that LPL 447X carriers appear to have a more favorable lipid profile and this allele appears to be a negative risk factor for coronary artery disease (CAD) [17-19]. An interesting recent report of neonatal somatic gene transfer with an adenoviral vector containing the S447X variant of LPL described rescue of 95% of LPL-deficient mice from lethality [20]. This study is further evidence of the gain of function of the S447X variant, as pointed out by Rader [21], because considerably fewer animals were rescued when the wild-type LPL vector had been used previously [22].

FCH is the most prevalent genetic lipid disorder observed in patients with CAD and their relatives, with a frequency of 1–2% in all populations examined and a prevalence of 10%-20% in patients with premature CAD [23-27]. Hypertriglyceridemia is a major component of the phenotype of FCH, the most common of the dyslipidemias [25-27]. The common metabolic defect in FCH appears to be hepatic overproduction of apolipoprotein B-containing TRL [28,29] and a preponderance of small dense low-density lipoprotein (LDL) particles [30,31]. Previously, we and others have proposed a genetic model for FCH that postulates the existence of both a common dominant major gene(s) that is further influenced by a number of modifier genes [23]. The identification of modifier genes could assist in finding the as yet undiscovered primary genetic determinant(s) by reducing the genetic heterogeneity of FCH [23].

Several reports have noted the presence of multiple lipoprotein phenotypes in obligate heterozygotes for LPL mutations that are reminiscent of FCH (i.e., hypertriglyceridemia, hypercholesterolemia, or both) [32]. As much as one-third of FCH patients have levels of post-heparin LPL activity and mass below the 10^th ^percentile for the general population [32,33]. Given the multifactorial nature of FCH, with other genetic or environmental factors (e.g., obesity) causing an increase in the hepatic production of lipoproteins, the catabolic capacity of LPL in individuals who are genetically predisposed to a low basal level of LPL activity may be overwhelmed, exacerbating the primary dyslipidemia and thus accelerating atherosclerosis [23]. Importantly, studies that have examined the role of genetic variation of the LPL gene in FCH have focused primarily on rare variations, mutations or intragenic markers [23,34-38].

The possibility that a disorder could be explained, at least in part, by either enrichment of pro-atherogenic genetic variations or depletion of anti-atherogenic ones led to our interest in examining the later in combined hyperlipidemia. The occurrence of impaired LPL activity in individuals with FCH suggested that examination of the anti-atherogenic LPL 447X allele could introduce a novel paradigm in the study of this disease. The aim of this study was to investigate the role of the LPL S447X polymorphism in a sample of subjects with combined hyperlipidemia and compare them with healthy controls. We provide evidence of a genetic association with LPL S447X with FCH and with lipoprotein composition in affected individuals.

## Results

### Characteristics of the study groups

Based Baseline on the hypothesis that a functional variation at the LPL gene locus would differ in frequency between dyslipidemic individuals and healthy controls, we screened patients and control subjects for the S447X polymorphism. The clinical characteristics of the two groups are described in Table 1. Individuals with combined hyperlipidemia displayed significantly elevated VLDL-TG (t = -15.310, 286.34 df, p < 0.001), LDL-TG (Z = -13.656, p < 0.001) and HDL-TG (t = -9.524, 383.36 df, p < 0.001) and decreased HDL-C (t = 10.010, 425.09 df, p < 0.001) compared to controls.

**Table 1:** Clinical characteristics and lipid analyses of the study population.

Trait	Controls (n = 161)	Cases (n = 212)	p-value
Age	46.8 ± 19.57 (161)	53.2 ± 12.08 (211)	<0.001
Gender(%female)	49.1 (161)	41.5 (212)	< 0.09
TC	183.9 ± 36.66 (161)	293.9 ± 72.83 (212)	*
TG	100.0 ± 37.81 (161)	376.7 ± 347.98 (212)	*
VLDL-C	11.1 ± 7.77 (133)	69.0 ± 57.90 (212)	*
VLDL-TG	47.7 ± 30.08 (133)	275.8 ± 256.25 (212)	<0.001
LDL-C	122.1 ± 31.08 (133)	180.5 ± 55.96 (211)	*
LDL-TG	29.9 ± 12.15 (133)	75.2 ± 177.92 (211)	<0.001
HDL-C	52.9 ± 14.18 (158)	40.6 ± 12.48 (212)	<0.001
HDL-TG	15.5 ± 4.88 (158)	21.7 ± 9.68 (212)	<0.001
TG/HDL-C	1.97 ± 1.42 (158)	11.46 ± 17.59 (212)	0.211
BMI	25.3 ± 4.08 (93)	27.7 ± 4.54 (154)	<0.001

With the exception of gender, means and standard deviations are reported. * Use of these variables as a case selection criterion precludes report of a statistic.

TC, total cholesterol; TG, triglyceride; VLDL-C, very low density lipoprotein – cholesterol; LDL-C, low density lipoprotein-cholesterol; HDL, high density lipoprotein; BMI, body mass index.

BMI was significantly increased in cases compared to controls (27.7 ± 4.54 vs. 25.3 ± 4.08; t = -4.170, p < 0.001 [95% CI: -3.53, -1.26]).

### Frequencies of the SNPs

Allelic and genotypic frequencies for S447X in cases and controls are listed in Table 2. The S447X genotype distribution did not deviate from Hardy-Weinberg expectations (χ^2 ^= 1.585; 2df; p = 0.453). It is noteworthy that the allelic and genotypic frequencies of the polymorphism in the control group were similar to previous reports [39,40].

Compared to healthy controls, the 447X allele frequency was significantly lower in cases (χ^2 ^= 29.085; 1df; p < 0.001; Odds Ratio = 0.192 with 95%CI: 0.096–0.378) (Table 2). Given the limited number of observations with respect to homozygous 447X subjects (n = 1), the XX and SX groups were collapsed for analysis. The genotypic distribution was significantly influenced by depletion of the X allele in controls versus cases (χ^2 ^= 26.087; 1df; p < 0.001; Odds Ratio = 0.191 with 95%CI: 0.098–0.371) (Table 2A). Though no significant gender differences were observed with respect to the S447X allelic or genotypic frequencies (Table 2B), male cases versus controls displayed a slightly greater depletion of the 447X allele (χ^2 ^= 19.738; 1df; p < 0.001; Odds Ratio = 0.145 with 95%CI: 0.059–0.355) than did female cases versus controls (χ^2 ^= 6.388; 1df; p = 0.011; Odds Ratio = 0.267 with 95%CI: 0.099–0.716). In the subset of subjects for whom BMI was recorded along with baseline lipid assessments (n = 247), no evidence of genetic association was observed in either cases (n = 154, SS:SX+XX, p = 0.216) or controls (n = 93, SS:SX+XX, p = 0.615).

**Table 2A:** Allelic and genotypic frequencies.

	Controls (n = 161)	Cases (n = 212)	p-value
447X	0.130	0.031	< 0.001
			
447 SS	0.745	0.939	
447 SX	0.248	0.061	
447 XX	0.006	0.000	
			
447 (SX+XX)	0.255	0.0.061	< 0.001

**Table 2B:** Allelic and genotypic frequencies, by gender.

	Controls		p-value	Cases		p-value
	Female (n = 79)	Male (n = 82)		Female (n = 88)	Male (n = 124)	

447X	0.1139	0.1463	0.485	0.0034	0.0028	0.950
						
447 SS	0.784	0.707	0.343	0.932	0.944	0.952
447 (SX+XX)	0.215	0.293		0.068	0.056	

447X, the minor allele for the lipoprotein lipase gene missense variation Serine447Stop (Ser447Ter, S447X); 447 SS, individuals homozygous for the Ser447 allele; 447 (SX+XX), individuals that carry either one or two 447X alleles.

### Genetic association of lipid parameters with S447X

To examine the effects of the polymorphism on lipoprotein metabolism, fasting lipoprotein concentrations provided a metabolic 'snap shot' for comparisons between carriers of the 447X allele and the 447S homozygotes. No significant changes in mean measures of plasma lipoprotein compartments were observed in the control group (Table 3). With the case group the S447 homozygotes displayed elevated VLDL-C (SS: SX+XX, Z = -2.003, p = 0.045) (Table 4). Suggestive evidence of an association with elevated VLDL-triglyceride (SS:SX+XX, Z = -1.883, p = 0.06) and elevated HDL-TG (SS:SX+XX, Z = -1.778, p = 0.075) in carriers of the 447X allele was also observed (Table 4).

**Table 3:** Plasma lipid concentrations in the control sample, grouped by LPL genotype.

		SS (n = 120)	SX + XX (n = 41)	p-value
Age		45.4 ± 18.69 (120)	50.8 ± 21.67 (41)	0.131

BMI		25.5 ± 4.38 (68)	25.0 ± 3.19 (25)	0.615
				
TG*	Total	92.1 ± 36.66 (120)	87.9 ± 41.30 (41)	0.544
	VLDL	47.8 ± 30.01 (98)	47.3 ± 30.70 (35)	0.934
	LDL	31.0 ± 12.30 (98)	27.0 ± 11.37 (35)	0.099
	HDL	15.7 ± 4.83 (117)	14.8 ± 5.00 (41)	0.303
				
C*	Total	184.8 ± 35.19 (120)	181.3 ± 41.01 (41)	0.654
	VLDL	11.3 ± 8.17 (98)	10.6 ± 6.59 (35)	0.796
	LDL	123.7 ± 27.00 (98)	117.5 ± 40.53 (35)	0.406
	HDL	52.6 ± 15.33 (117)	53.9 ± 10.34 (41)	0.553
				
TG/HDL-C		2.05 ± 1.51 (117)	1.75 ± 1.08 (41)	0.211

*Lipid measurements expressed in mg/dL. Total cholesterol assessed by Wilcoxon two sample test.

TC, total cholesterol; TG, triglyceride; VLDL-C, very low density lipoprotein – cholesterol; LDL-C, low density lipoprotein-cholesterol; HDL, high density lipoprotein; BMI, body mass index; SS, individuals homozygous for the Ser447 allele; SX+XX, individuals that carry either one or two 447X alleles.

**Table 4:** Plasma lipid concentrations in the combined hyperlipidemia subjects, grouped by LPL genotype.

		SS (n = 199)	SX + XX (n = 13)	p-value
Age		53.3 ± 12.28 (198)	51.7 ± 8.70 (13)	0.647

BMI		27.6 ± 4.60 (145)	29.6 ± 3.21 (9)	0.216
				
TG*	Total	383.8 ± 357.69 (199)	267.2 ± 65.24 (13)	0.099
	VLDL	281.7 ± 262.98 (199)	184.6 ± 62.40 (13)	0.060
	LDL	76.3 ± 183.55 (199)	59.4 ± 24.22 (13)	0.809
	HDL	21.7 ± 8.72 (199)	21.9 ± 19.84 (13)	0.075
				
C*	Total	295.5 ± 73.96 (199)	269.2 ± 48.10 (13)	0.264
	VLDL	70.4 ± 59.29 (199)	48.4 ± 21.43 (13)	0.045
	LDL	180.8 ± 56.74 (199)	175.3 ± 43.55 (13)	0.926
	HDL	40.4 ± 11.87 (199)	43.7 ± 20.09 (13)	0.661
				
TG/HDLC		11.70 ± 18.07 (199)	7.82 ± 5.849 (13)	0.153

* Lipid measurements expressed in mg/dL. All test statistics are non-parametric (Wilcoxon two sample test).

TC, total cholesterol; TG, triglyceride; VLDL-C, very low density lipoprotein – cholesterol; LDL-C, low density lipoprotein-cholesterol; HDL, high density lipoprotein; BMI, body mass index; SS, individuals homozygous for the Ser447 allele; SX+XX, individuals that carry either one or two 447X alleles.

### Linear regression analysis of the effects of S447X on lipid measures

Regression models for lipid measurements included a data-driven selection among the polymorphism category SS versus SX+XX. Potential predictors included age, gender, and clinical category (case or control). Models in which a polymorphism effect did not achieve p < 0.10 are not reported. Among the lipid measures, a logarithmic transform was found to be generally appropriate for use in the models. No significant models were indicated for TC, VLDL-C, or VLDL-, LDL-, and HDL-triglyceride. For transformed lipid measures, estimated adjusted means on the original scale are presented (Table 5).

**Table 5:** Estimated adjusted means for plasma concentrations of plasma lipids, grouped according to *LPL *genotype.

Trait	n	SS*	SX+XX*	R^2^	p-value†
TG	373	165.8	146.1	0.697	0.001
LDL-C	345	144.5	133.4	0.309	0.007
TG/HDL-C	370	3.801	3.162	0.651	0.031

* Lipid measurements expressed in mg/dL. ^†^The p-values are for the corresponding adjusted means on the log scale.

TG, triglyceride; LDL-C, low density lipoprotein-cholesterol; HDL, high density lipoprotein; SS, individuals homozygous for the Ser447 allele; SX+XX, individuals that carry either one or two 447X alleles.

For total triglycerides (n = 372), the best model selected SX+XX vs. SS (p = 0.001). Selected effects include gender (p = 0.001), clinical category (p < 0.001) and genotype (p = 0.062). The adjusted R-square for the model was 0.697 (Table 5). The group of S447 homozygotes displayed modestly elevated plasma triglycerides compared with carriers of the 447X allele (+19.7 mg/dL). For LDL-C (n = 345), the best model selected was also SX+XX vs. SS (p = 0.007). Selected effects included clinical category (p < 0.001) and genotype (p = 0.087). The adjusted R-square for the model was 0.309 (Table 5). The group of S447 homozygotes displayed modestly elevated LDL-C as compared with carriers of the 447X allele (+11.1 mg/dL). For the ratio of total triglycerides to HDL-C (n = 370), the best model selected was again SX+XX vs. SS (p = 0.031). Selected effects include gender (p = 0.001), clinical category (p < 0.001) and genotype (p = 0.044); the adjusted R-square for the model was 0.651 (Table 5). The group of S447 homozygotes displayed modestly elevated ratio as compared with carriers of the 447X allele (+0.639).

Though BMI measurement was available on all subjects, a measure of BMI at the same time point as the baseline lipoprotein measurement was not available for all subjects. Regression models including BMI resulted in unacceptably small counts in cells, and thus analysis of the subset including BMI was not reported.

## Discussion

The common S447X truncation polymorphism of LPL is associated with a cardio-protective lipid profile and a modest reduction in risk for CAD [17]. Data from this study support the hypothesis that there is significant depletion in allelic and genotypic frequencies for this protective polymorphism in individuals with combined hyperlipidemia compared to healthy controls. Specifically, we found that 447X exists in 13% of healthy controls but only in 3% of cases (p < 0.001). The frequency of 447X in the control group is similar to that in the Framingham Offspring Study that examined 1114 men and 1144 women and found the frequency of the S447X variant was 16 and 17.5% in men and women, respectively [19]. As in a previous report [19], data from this study showed no significant gender differences with respect to the LPL allelic or genotypic frequencies, though male cases versus controls displayed a slightly greater depletion of the 447X allele than did female cases versus controls.

Several population studies have reported cardio-protective alterations in lipoprotein profiles in subjects who carry the 447X allele (e.g., lower TRL, lower VLDL-C, higher apo AI levels, higher HDL-C, protection against CHD) [5,7,41-44]. The linear regression analysis in this study showed that carriers of the 447X allele displayed modestly elevated plasma triglycerides (+19.7 mg/dL), LDL-cholesterol (+11.1 mg/dL), and ratio of TG over HDL-C (+0.639) as compared with carriers of the 447S allele. These results are congruent with population studies of cardiovascular disease that have reported similar effects on TG [4,45], increased HDL-cholesterol, and 0.8-fold reduced risk of ischemic heart disease in 447X carriers. Although associations with favorable changes in both VLDL-C [42] and HDL-C have been reported, no association between LDL-C and this polymorphism has been demonstrated previously. We believe that it is likely that the case selection criterion we employed (elevated LDL-C) could potentially account for the association between the 447X allele and lower LDL-C. Although another polymorphism within LPL has been associated with alterations in the TG/HDL-C ratio [46], no study has previously explored the impact of this polymorphism on the ratio. However, one may infer that similar findings were demonstrated in the longitudinal analysis of the Bogalusa Heart Study (i.e., increased frequency of 447X in subjects in the bottom quartile for plasma TG and HDL-C) [41].

Though several studies have examined the co-segregation of genetic variations within the LPL gene region with the occurrence of FCH [23], the few that have examined the role of the 447X allele lacked sufficient power to evaluate the significance of this polymorphism in FCH. Of the four studies that examined the S447X polymorphism in 31 [47], 40 [36], 20 [37], and 30 [34] unrelated probands, respectively, only Campagna and colleagues [34] reported a modest effect of the S447X polymorphism on lipid levels. Given the frequency of this polymorphism and the sample sizes of these aforementioned studies, detection of the genetic association reported here would not be expected.

Potential study limitations include a possibility of selection bias of combined hyperlipidemia subjects without the use of familial primary dyslipidemia criterion. A goal of our group is to identify an endophenotype that would allow the identification of individuals with FCH without the resource intensive assessment of relatives. However, it is important to note that our group has successfully validated several genetic associations reported for FCH [48,49]. Another potential limitation involved the availability of BMI, an index known to impact lipoprotein metabolism and lipid homeostasis [46,50], in the entire study sample for inclusion in regression analyses.

### Summary

As one would expect in a multifactorial model of a common disease, contributive alleles would be expected to add an incremental risk of disease (i.e., CAD). Common deficiencies in LPL may be the underlying causes of significant increases in CAD risk [1,2]. Understanding the role that functional gene polymorphisms play in risk and determining the levels of intermediate phenotypes (e.g., TG) is essential to our understanding of the important metabolic pathways in the diseased and disease-free state. Given the prevalence of the LPL S447X polymorphism in the population, greater knowledge of the underlying consequences of this variation may be of considerable importance in understanding genetic predisposition to atherosclerosis and heart disease [51]. S447X is a common, functional variant and is associated with a beneficial lipid profile, and is significantly depleted in patients with combined hyperlipidemia. Importantly, understanding the contribution not only of susceptibility alleles in dyslipidemia, but also of depletion of protective alleles, may contribute materially to the identification of the genetic determinants of heart disease.

## Methods

### Study design

This study was a retrospective analysis of the prevalence of a common LPL polymorphism in non-Hispanic Caucasian (European-descent) subjects with combined hyperlipidemia and a control group. Subjects were selected from the University of California, San Francisco (UCSF) Genomic Resource in Arteriosclerosis [52], using the following criteria: combined hyperlipidemia with total plasma cholesterol (TC) > 200 mg/dL, total plasma TG > 200 mg/dL, LDL-C > 130 mg/dL, and very low density lipoprotein-cholesterol (VLDL-C) > 30 mg/dL. Exclusion criteria were: a diagnosis of familial hypercholesterolemia, hypothyroidism, or Type 2 Diabetes Mellitus. Healthy non-Hispanic Caucasian (European-descent) subjects with normal lipoprotein profiles comprised the control group. All subjects gave informed consent in a protocol approved by the UCSF Committee on Human Research. With the exception of Body Mass Index (BMI, n = 247) clinical and demographic data were available on all subjects (n = 373).

#### Genotypic and phenotypic studies

Genomic DNA was prepared from whole blood and was drawn after a 10-hour fast [52]. VLDL were prepared by ultracentrifugation [53]. HDL-C was measured after precipitation of apo-B-containing lipoproteins with dextran sulfate and magnesium [54]. Cholesterol and TG levels were measured in plasma and in lipoprotein fractions by either automated fluorescence method or automated chemical analysis [55]. LDL-C was calculated as TC minus HDL-C plus VLDL-C. Standards were provided by the Centers for Disease Control (Atlanta, Georgia, USA). Baseline lipoprotein measurements were obtained when patients had received no lipid lowering medication for at least 1 month. The presence or absence of the S447X polymorphism was determined as described previously [17].

### Statistical methods

The SPSS for Windows (v11.0.1, 2001) system for statistical analysis was used. Allele and genotype frequencies were determined by the gene-counting method. Tests for Hardy-Weinberg equilibrium in controls, and allelic or genotypic association in cases versus controls, were evaluated by χ^2 ^test. χ^2 ^tests of allele frequency were adjusted using Yates' Continuity Correction. Power transformations of potential predictor variables were examined where appropriate. Two-group comparisons of means of transformed or normally distributed variables used the independent samples t-test. Two-group comparisons of means of untransformed, non-normally distributed variables used the Wilcoxon two-sample test. The procedure, general linear model (GLM), was used for linear regression models. Power transformations of potential predictor variables were examined to maximize the explanatory power of the overall model (by maximizing the F statistic). Interactions between covariates and genotypes were evaluated. Selected interaction effects and covariate-adjusted means of the transformed responses for levels of categorical factors were tested using procedure GLM. Interaction effects with p < 0.10 were retained. For multiple comparisons between factor levels, Bonferroni-corrected p-values are reported.

## Competing interests

The author(s) declare that they have no competing interests.

## Authors' contributions

SFW carried out genetic analyses, participated in the statistical analysis and coordinated the preparation of the manuscript. MVK carried out genetic analyses. CRP managed the genomic resource from which study subjects were identified, provided specimens for analysis and participated in manuscript preparation. MJM recruited study subjects and participated in the study design and manuscript preparation. JPK recruited study subjects and participated in the study design and manuscript preparation. BEA conceived of the study, participated in its design and coordination, statistical analyses, and participated in manuscript preparation.
